# Death at the interface: Nanotechnology’s challenging frontier against microbial surface colonization

**DOI:** 10.3389/fchem.2022.1003234

**Published:** 2022-10-13

**Authors:** Kiran Deep Kaur, Olivier Habimana

**Affiliations:** ^1^ The School of Biological Sciences, The University of Hong Kong, Hong Kong, Hong Kong SAR, China; ^2^ Guangdong Technion Israel Institute of Technology (GTIIT), Shantou, Guangdong, China

**Keywords:** biomimetics, antimicrobial surfaces, surface nano/microstructures, functional surface coating, antimicrobial-resistant microorganisms

## Abstract

The emergence of antimicrobial-resistant bacterial strains has led to novel approaches for combating bacterial infections and surface contamination. More specifically, efforts in combining nanotechnology and biomimetics have led to the development of next-generation antimicrobial/antifouling nanomaterials. While nature-inspired nanoscale topographies are known for minimizing bacterial attachment through surface energy and physicochemical features, few studies have investigated the combined inhibitory effects of such features in combination with chemical alterations of these surfaces. Studies describing surface alterations, such as quaternary ammonium compounds (QACs), have also gained attention due to their broad spectrum of inhibitory activity against bacterial cells. Similarly, antimicrobial peptides (AMPs) have exhibited their capacity to reduce bacterial viability. To maximize the functionality of modified surfaces, the integration of patterned surfaces and functionalized exteriors, achieved through physical and chemical surface alterations, have recently been explored as viable alternatives. Nonetheless, these modifications are prone to challenges that can reduce their efficacy considerably in the long term. Their effectiveness against a wider array of microbial cells is still a subject of investigation. This review article will explore and discuss the emerging trends in biomimetics and other antimicrobials while raising possible concerns about their limitations and discussing future implications regarding their potential combined applications.

## Introduction

The ubiquitous nature of microbes is linked with their ability to adapt and survive on most biotic and abiotic surfaces in our biosphere. These microbes typically exist and thrive as either adhered single cells or part of a complex consortium of microorganisms. Because of their genetic flexibility, a broad spectrum of microorganisms has developed to adapt to various stressful environments, especially those subjected to antimicrobial agents that prevent their survival and further proliferation. The inherent genetic flexibility and adaptability of microbial populations have allowed these microbial populations to develop antimicrobial resistance through an accumulation of favourable mutations, particularly at specific target sites, over time or by acquiring novel biological functions (Wang & Vermerris, 2016), consequently leading to the emergence of antimicrobial-resistant bacterial strains. Modern medical treatments heavily depend on antibiotics to control infections and allow for invasive surgery. The emergence of antibiotic-resistant bacteria poses a considerable threat to the survival of affected individuals. In addition, undesirable bacterial colonization of surfaces can promote the formation of biofilms. Biofilms are colonies of microorganisms enclosed in extracellular polymeric substances (EPS) attached to the surface (Zhang & Wagner, 2017). Biofilm development is prevalent and holds immense significance to bacterial communities. Biofilms are known to be able to form on solid or liquid surfaces and tissues of living beings (McKeen, 2012). Occupation of a surface by planktonic microbes is followed by irreversible attachment of bacteria to the surface, an event which is generally preceded by reversible attachment of the microbes. Cellular division and aggregation form microcolonies, which mature over time to form robust biofilms (Zhang & Wagner, 2017). Several characteristic factors govern the rate and the extent of the development of biofilms, such as physical and chemical characteristics of the surface, for instance, charge, temperature, pH and roughness of the surface, external physical, chemical or biological stimuli from the microenvironment, quorum sensing and nutrient limitations (Zhang & Wagner, 2017; Alotaibi & Bukhari, 2021). Bacteria bound to biofilms are more resistant to antibiotic and antiseptic treatments (Chen et al., 2013). The surrounding extracellular polymeric matrix enhances the survival capacity of these microorganisms in hostile environments. It is believed that the innate resistance is conferred by a shift in metabolism, the creation of a physical barrier through an encasement of biofilm-bound microbes in a protective ‘shield’ of EPS and the upregulation and exchange of genes responsible for producing antimicrobial resistance (Zhang & Wagner, 2017). Hence, strategies that can effectively prevent microbes’ adhesion to avoid subsequent biofilm development are currently being explored and developed.

Technological advances, particularly nanotechnology, have led to the emergence of practical techniques that can be manipulated to create antimicrobial/antifouling surfaces (Jaggessar et al., 2017). These antimicrobial surfaces possess physical features that can potentially hinder the attachment of bacteria onto a given surface, preventing colonization and potential biofilm formation. Antimicrobial surfaces can be either anti-fouling surfaces with the potential to prevent bacterial attachment/adhesion or bactericidal surfaces, which kill bacteria upon contact upon attachment (Wang & Vermerris, 2016). One of the key features of antifouling surfaces is their low surface energy and high surface contact angle, which confer super-hydrophobicity and anti-wetting properties to a given surface. Super-hydrophobicity has been hypothesized to be a prerequisite to anti-fouling by reducing the contact area between bacteria and nanostructured surfaces, consequentially preventing adhesion from forming an effectively repellent surface (Wang & Vermerris, 2016; Mahanta et al., 2021). Generating nanoscale roughness with materials with low surface energy is the key to constructing superhydrophobic surfaces. Roughness at the nanoscale can be achieved by creating biomimetic nanopillars or grooves spanning the surface, through which bacterial adhesion and colonization can be impeded by reducing surface contact area. The spacing, size and aspect ratio of these nanostructures considerably influences the effectiveness of their antimicrobial surfaces, which can determine their antimicrobial potential. Antimicrobial nanomaterials are recognized by their large surface-area-to-volume ratio (Wang & Vermerris, 2016). Nanoscale roughness significantly reduces contact area and adhesion area.

Natural surfaces of lotus leaves, shark skin, gecko skin/feet and wings of cicada, dragonfly and butterfly have been identified as possessing topographies that exhibit low surface energy and high surface contact angle and, hence, antimicrobial functions. Bio-inspired surface engineering has emerged to create a new class of antimicrobial surfaces exhibiting similar topographies in recent years. Integrating topographies inspired by these natural surfaces with nanotechnology has enabled the construction of nanomaterials mimicking the antimicrobial/anti-fouling properties of natural surfaces. Whether in their natural form or engineered, these surfaces’ antimicrobial potential is typically determined by their geometrical properties. In one instance, dermal denticles across the surface of shark skin form a rough texture reducing the adhesion area available to aquatic organisms, including microbes (Kanagusuku et al., 2021). The significant reduction in adhesion area results in a self-cleaning capacity of shark skin. In contrast, the curved spinules on gecko skin create a superhydrophobic, resulting in a high surface contact angle, and repellent physical barrier with considerably low surface energy, allowing droplets of fluid to roll off effortlessly. Cell adjustment to adapt to nanoscale topography is thermodynamically unfavourable as it requires immense energy expenditure. Hence, manipulating surface topographies can prevent bacterial adhesion without causing the bacteria to develop resistance.

One alternative to producing antimicrobial surfaces is the creation of functionalized antimicrobial nanofiber coatings (Wang & Vermerris, 2016). The design of nanofibers has become possible with the development of electrospinning, template synthesis and self-assembly. Electrospinning is the process by which charged threads of polymer solutions or melts can produce diameters on the nanometer scale. Biopolymers have received much attention for their potential role as antimicrobial surfaces. Chitosan, cellulose and antimicrobial peptides (AMPs) have exhibited desirable antimicrobial functions. Through physical immobilization on surfaces using various techniques such as layer-by-layer deposition, AMPs have demonstrated their ability to reduce concentrations of microbes after incubation (Lei et al., 2019). In creating antimicrobial surfaces, the polymers are typically positively charged to facilitate electrostatic interactions of polycationic polymers with the anionic exterior of bacterial cells (Lei et al., 2019). These interactions attract bacterial cells to the polycationic polymer surface, facilitating cell-to-surface adhesion interactions. As a result, bacterial cell membranes are disrupted, and vital intracellular components leak out of the cell, causing cell lysis. The hydrophobicity of AMPs promotes their penetration into the hydrophobic interior of the cell membrane of bacterial cells, resulting in the destabilization of the bacterial cell membrane. Bio-coatings remain attractive as they do not leach into the surrounding medium (Cyphert & von Recum, 2017).

Chemical processes of surface modification by introducing Quaternary Ammonium Compounds (QACs) to target cationic polymer surfaces as potential antimicrobial agents have also been used traditionally (Xue et al., 2015). These compounds can be incorporated into polymer nanofibers to enhance their antimicrobial functions. QACs are desirable due to their environmental stability, impressive cell membrane penetration properties, relatively low toxicity and corrosivity (Roy et al., 2008). The antibacterial activity of QACs heavily depends on their overall molecular structure and the length of their alkyl chain (Roy et al., 2008). The non-selective nature of QACs enhances their ability to target a broad spectrum of microorganisms regardless of their overall membrane structure, making it possible to use against a wide range of bacteria (Orlando et al., 2020).

With the emergence of a wide array of chemical and physical techniques for creating antibacterial and antifouling surfaces, it has become increasingly important to assess the efficacy and effectiveness of these methods in terms of functionality and the level of protection provided while minimizing any possible risks and maximizing durability. Though the antimicrobial functions of chemically modified surfaces are notable, chemical modifications may not be able to entirely reduce the chances of toxicity or degradation in the longer run. However, physical changes alone may also be insufficient in providing maximum protection against microbial colonization, owing to the complexity of bacterial cell wall interactions with topological nanostructures and the possibility of bacterial infiltration of narrow spaces between the nanostructures. This has created the need to explore more potent mechanisms of surface modification that specifically target the vital characteristics/functions of bacterial cells, particularly those involved in biofilm formation, motility and pathogenesis. Bacterial cells rely primarily on quorum sensing to achieve major physiological processes. Several novel antimicrobial surfaces and agents are currently being explored because of the need to target specific cell signaling mechanisms to hinder essential physiological functions in bacterial cells and biofilm production. Apart from developing antimicrobial surfaces containing AMPs and QACs, some current potential methods include using quorum sensing inhibitors (QSIs), such as natural or synthetic furanone, and antimicrobial enzyme multilayer coating facilitated by enzyme immobilization.

Likewise, switchable surfaces with dual functions have also been explored in recent studies to maximise the antimicrobial effects of modified surfaces. The physicochemical features of these surfaces have been meticulously exploited to attain antifouling and bactericidal properties. Harnessing these surfaces for the desired purpose involves inducing changes, either in the temperature or the pH, in the surrounding environment, i.e. aqueous solution, which consequentially triggers a conformational change of nanopatterned polymer brushes to expose the underlying substrate layer of adsorbed antibiotic agents (Yu et al., 2014b; Yan et al., 2016; Mahanta et al., 2021). Initial temperature changes or pH changes can cause these polymer brushes to bend down and facilitate contact between bactericidal agents, like AMPs, enzymes, QACs etc., and bacterial cells (Yu et al., 2014b; Yan et al., 2016; Wang et al., 2018). Depending on the conformation of these nano-polymers, these surfaces can potentially kill and eliminate bacterial cells. Nonetheless, research on these self-cleaning surfaces is limited.

**FIGURE1:**
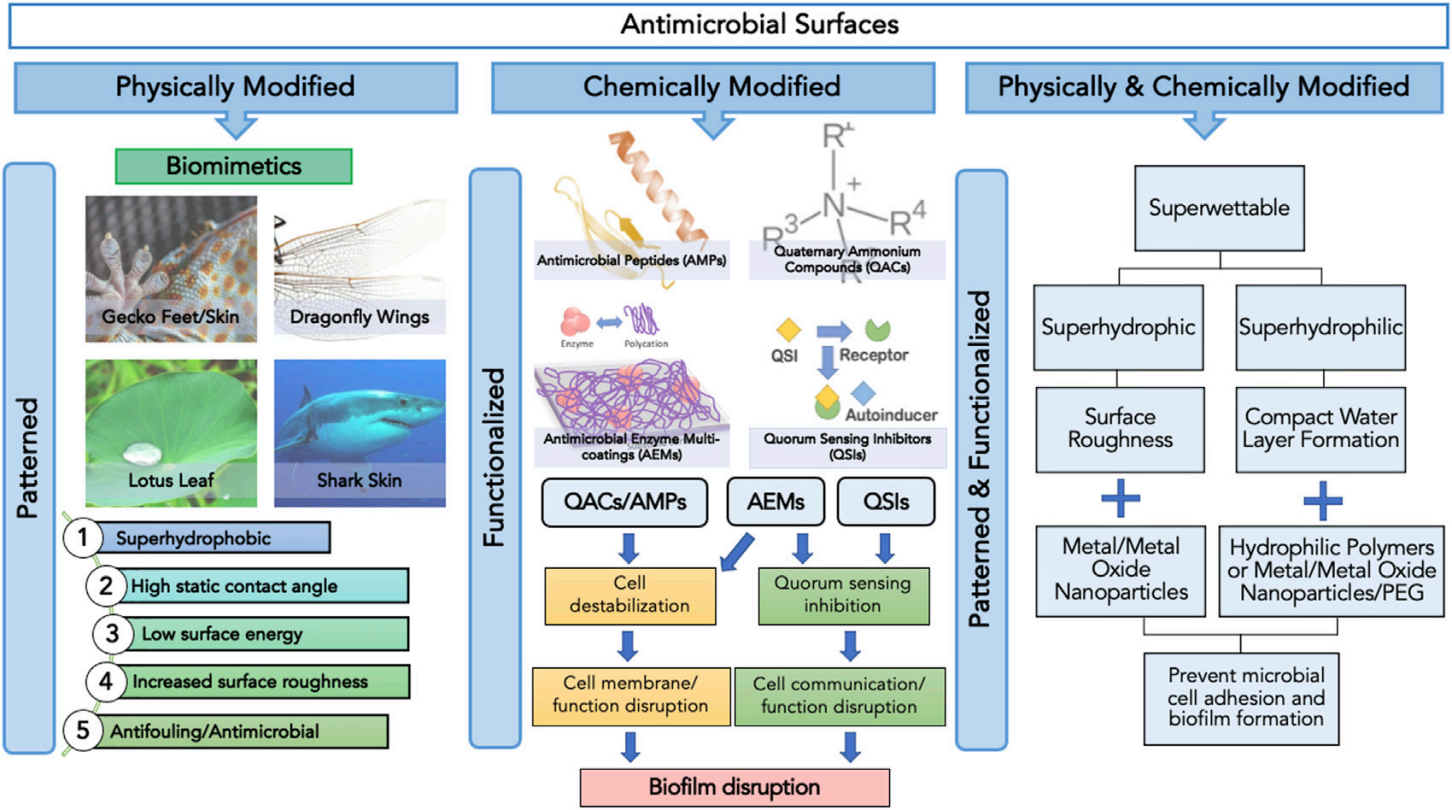
Categories of antimicrobial surfaces based on the method of surface modification, namely physical, chemical and physicochemical, and their respective surface features and functions.

This review article will discuss the application and limitations of emerging trends in biomimetics and other antimicrobials against microbial adhesion and surface colonization while discussing future implications regarding their potential combined applications (Figure 1).

## Antibacterial biomimetics and bio-coatings with biofilm inhibition activity

### Emerging trends in biomimetics

Surfaces are typically covered with various microbes, some of which may potentially result in detrimental health complications. Consequently, hygienic/aseptic measures have traditionally made use of antimicrobial agents, which at times are limited or inefficient due to their surface-sensitive/specific properties or the difficulty in accessing hard-to-reach targeted areas (Wang & Vermerris, 2016). The need to minimize bacterial growth on critical surfaces has given rise to demands for creating antimicrobial characters that would potentially reduce bacterial attachment, the precursor of biofilm formation. Surprisingly, nature has drawn appreciable inspiration in developing such antimicrobial surfaces.

The antimicrobial and antifouling effects of an impressive array of natural nanoscale topographies have inspired the creation of a new generation of engineered surfaces through physical modifications of surface topographies (Vellwock & Yao, 2021). The construction and characterization of nanoscale materials and structures with enhanced physical and chemical functional properties lie at the heart of nanotechnology advances (Wang & Vermerris, 2016). This has expedited the emerging trend of biomimetics with antimicrobial and antifouling properties (Nir & Reches, 2016). Natural surfaces of lotus leaves, shark skin, gecko skin/feet and wings of cicada, dragonfly and butterfly are well known to possess topographies that exhibit physical features characterized by their high surface-area-to-volume ratio and super-hydrophobic properties.

**FIGURE 2:**
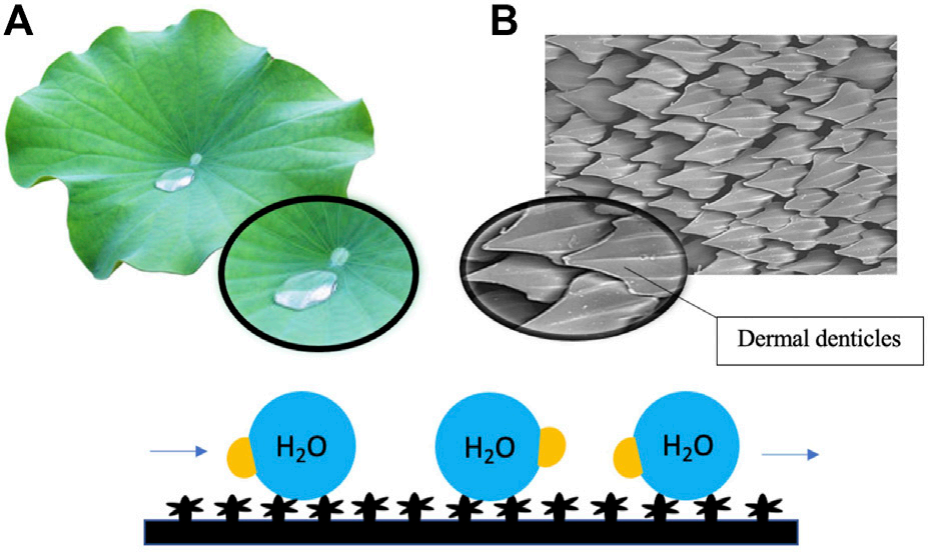
Surface roughness induced by hierarchical micro- and nanostructures of lotus leaves **(A)** and shark skin **(B)** renders the surface superhydrophobic and low in surface energy, resulting in the repulsion of water droplets and reducing adhesive forces between settling bacteria and the surface. Dirt particles and debris are picked up by flowing water droplets on the surface.

For instance, the antifouling potential of lotus (*Nelumbo nucifera*) leaves is understood to lie in the intrinsic physical features of the leaf surface, which have binary structures at both microscale and nanoscale (Wang & Vermerris, 2016). The hierarchical surface structure built by randomly oriented small hydrophobic wax tubules on the top of convex cell papillae confers hierarchical roughness to lotus leaves, enabling the superhydrophobic surface to attain more excellent stability (Nosonovsky & Bhushan, 2007; Koch et al., 2009). The innate structure of lotus leaves engenders a phenomenon recognized as the ‘lotus effect, which refers to the self-cleaning properties of lotus leaves caused by the ultra-hydrophobic nature of the leaf’s surface. The lotus effect enables the trapping of proportionally large amounts of air and, as a result, minimizes the contact area between water droplets and the surface (Wang & Vermerris, 2016). Dirt particles are picked up by water droplets, which do not disperse due to the high surface tension of water and the leaves’ superhydrophobic, microscopic and nanoscopic architecture (Li & Guo, 2019) (Figure 2). The microstructure of leaves with superimposed nanostructure of hydrophobic waxes results in high static contact angle, low tilt angle and common contact angle hysteresis, which in turn contribute to the antifouling properties of lotus leaves (Koch et al., 2009). Low surface energy reduces adhesive forces between settling bacteria and the surface, enabling bacteria removal before biofilm formation. In recent studies, the topography of lotus leaves has inspired the creation of artificial biomimetic surfaces with similar anti-fouling characteristics. In one well-noted approach, the hierarchical structure of the lotus leaf was recreated by moulding lotus leaves and self-assembling the natural lotus wax deposited by thermal evaporation to form wax tubules nanostructures to construct micro-structured lotus leaf replicas (Koch et al., 2009). Hierarchical structures of lotus leaves were replicated by combining micro and nanostructures. The formulated lotus leaf replica possessed features and functions almost identical to a natural lotus leaf.

Similarly, the antifouling properties of shark skin have also led to the development of shark skin-inspired biomimetics. Sharkskin is characterized by micron-sized grooved scales, known as dermal denticles, that grow on the skin and interlock to form a naturally rough surface (Liu & Li, 2012). It has been reported in recent studies that the grooved scales along the axis of the body can change the flow of water and reduce drag, thereby decreasing contact between microbes and the skin surface (Chien et al., 2020). The presence of dermal denticles across the skin’s surface forms a rough texture, which reduces the adhesion area available to aquatic organisms, including microbes. The significant reduction in adhesion area results in the self-cleaning capacity of shark skin (Figure 2). A combined effect of surface hydrophobicity, resulting in water repellency and innate roughness, results in the antifouling effect of shark skin. It is currently believed that coatings with topographical features smaller than the parts of the organisms on the surface or the dimension of the organism itself can be more effective as an antifouling surface (Banerjee et al., 2011). Furthermore, more densely packed denticles with larger widths and higher concave grooves can increase hydrophobicity (Chien et al., 2020). Manipulation of nanotechnology to construct shark skin-inspired biomimetics is manifested in the creation of sharklet, a commercial plastic sheet product with unique microbe-resistant properties inspired by these overlapping, ridged platelet structures of shark scales (Liu & Li, 2012; Chien et al., 2020). Variations in surface topography impose stress gradients on the surface membrane of the settling microorganisms during the initial contact, disrupting normal cell functions and forcing the microorganism to provide energy to equalize the stress through adjustment of each topological feature (Zhang & Wagner, 2017). The persistent mechanical stress inflicted upon the settling bacteria by the topography of the sharklet is thermodynamically unfavourable to the settling cell as adjustment requires an undesirable expenditure of energy.

Next-generation mechano-bactericidal surfaces of varying degrees of surface hydrophilicity/hydrophobicity have also demonstrated their capacity to impose mechanical stress on settling bacterial cells, thereby inducing cell rupture and death (Ivanova et al., 2020; Linklater et al., 2020; Valiei et al., 2022). Based on their physical characteristics, mechano-bactericidal surfaces have been classified into two major categories, one containing nanopillars, with heights of 220, 360, and 420 nm, and the other sharp nano-edges (Ivanova et al., 2020; Linklater et al., 2020). The bactericidal activity is influenced mainly by the elasticity and orderly positioning of the nanopillar protrusions (Ivanova et al., 2020). Upon settlement onto the surface, bacterial cells come into close contact with nanopillars protruding upwards which induces conformational changes in bacterial cells. The structural changes induced by the physical pressure exerted on the cells pose significant stress on the bacterial cell membrane, causing it to stretch to the extent that causes cell membrane disruption and tearing of the cell. The mechanical bactericidal action was first evidenced using dragonfly nanopillars, which were shown to be caused by mechanical stress through strong adhesive forces (Bandara et al., 2017), and later substantiated through elastic energy estimation upon interaction by bacteria (Bandara et al., 2020). Subsequent biomimicry inspired studies reported on the bactericidal activity of silicon or titanium nanopillar arrays, highlighting their profound killing effect on bacterial cells. Ivanova et al. have concluded a higher degree of bacterial cell death induced by silicon nanopillars, ideally at 360 nm height, which increases nanopillar elasticity, of Gram-negative *Pseudomonas aeruginosa* than Gram-positive *Staphylococcus aureus* (Ivanova et al., 2020). Examination of the mechano-bactericidal impact of silicon nanopillars in another study suggested an increase in the fatality of *P. aeruginosa* bacterial cells in the absence of a wet surface layer or the presence of air bubbles on the nanopillars, thereby proposing that super hydrophilicity provides a more favourable killing surface (Valiei et al., 2022). Nano-edges, like graphene sheets, on the other hand, rely on their lipophilic nature to attract the hydrophobic lipid tails in the cell membrane and, as a result, detach lipids from the phospholipid bilayer, causing hole formation in the bacterial membrane and cell content leakage (Linklater et al., 2020).

The bactericidal effects of these artificial nanostructures are comparable to the similar effects of cicada wings. The cicada wing comprises chitin, a fibrous polysaccharide, and a diverse array of proteins and wax (Ivanova et al., 2012; Shahali et al., 2019). Nanopillars spanning across the surface of the wing complete the surface architecture of the wings (Shahali et al., 2019; Linklater et al., 2020). The dimensions of nanopillars on the cicada wing surface occur in a wide range. The diameter and height of nanopillars have been revealed to range from 82 to 148 nm, and 146–159 nm, respectively, with pillar spacing ranging between 44 and 177 nm (Ivanova et al., 2012; Kelleher et al., 2015). Closely packed tall nanopillars arranged in an orderly manner determine the hydrophobicity of cicada wings, the degree of which can increase as the contact angle of the wings increases in the presence of a wax layer (Sun et al., 2009). Studies have suggested that the antimicrobial action of cicada wings is comparatively more effective on Gram-negative bacteria owing to the elasticity of the bacterial membrane, facilitating structural changes due to mechanical stress (Hasan et al., 2012). In contrast, Gram-positive bacteria have rigid membranes which confer resistance to the fatal effects of cicada wings. *Escherichia coli* and *P. aeruginosa* have shown considerable susceptibility to the antimicrobial effects of cicada wings (Pogodin et al., 2013). These observations are consistent with many studies on nanopillar constructions inspired by cicada wings.

The benefits of physical surface modification methods are noteworthy by allowing for greater and more precise control over the surface modification process and reducing the propensity of microbes to develop antimicrobial resistance. These methods do not require extensive alteration of compounds of interest to achieve surface modifications, hence, maintaining the efficacy and antimicrobial activity of the surface, as opposed to chemical processes. Nonetheless, physical approaches alone may also not be entirely sufficient in providing the ultimate protection against the threat of microbial colonization. The fact that biofilms, in essence, are the most efficient means of bacterial survival in the environment highlights the need to develop more elaborate methods of curbing bacterial colonization and, consequently, biofilm formation. Therefore, it can be expected that these microorganisms will always find ways to colonize surfaces as means of survival. In addition, possibilities of bacterial infiltration on physically modified surfaces cannot be completely ruled out, as bacteria may penetrate the narrow sites between the biomimetic nanoparticles and, thereby, proliferate, despite the repulsive and biocidal nature of these surfaces. Also, physically modified surfaces may not necessarily be completely sustainable without ‘self-cleaning’ mechanisms. The accumulation of dead bacteria on these antimicrobial surfaces may promote bacterial colonization, resulting in reduced antimicrobial activity in the longer term.

### Antibacterial quorum sensing inhibitors

Owing to the physical and chemical modifications of surfaces, novel mechanisms that can disrupt critical regulators of bacterial cell-to-cell communication need to be incorporated to enhance the long-term effectiveness of antimicrobial surfaces.

**FIGURE 3:**
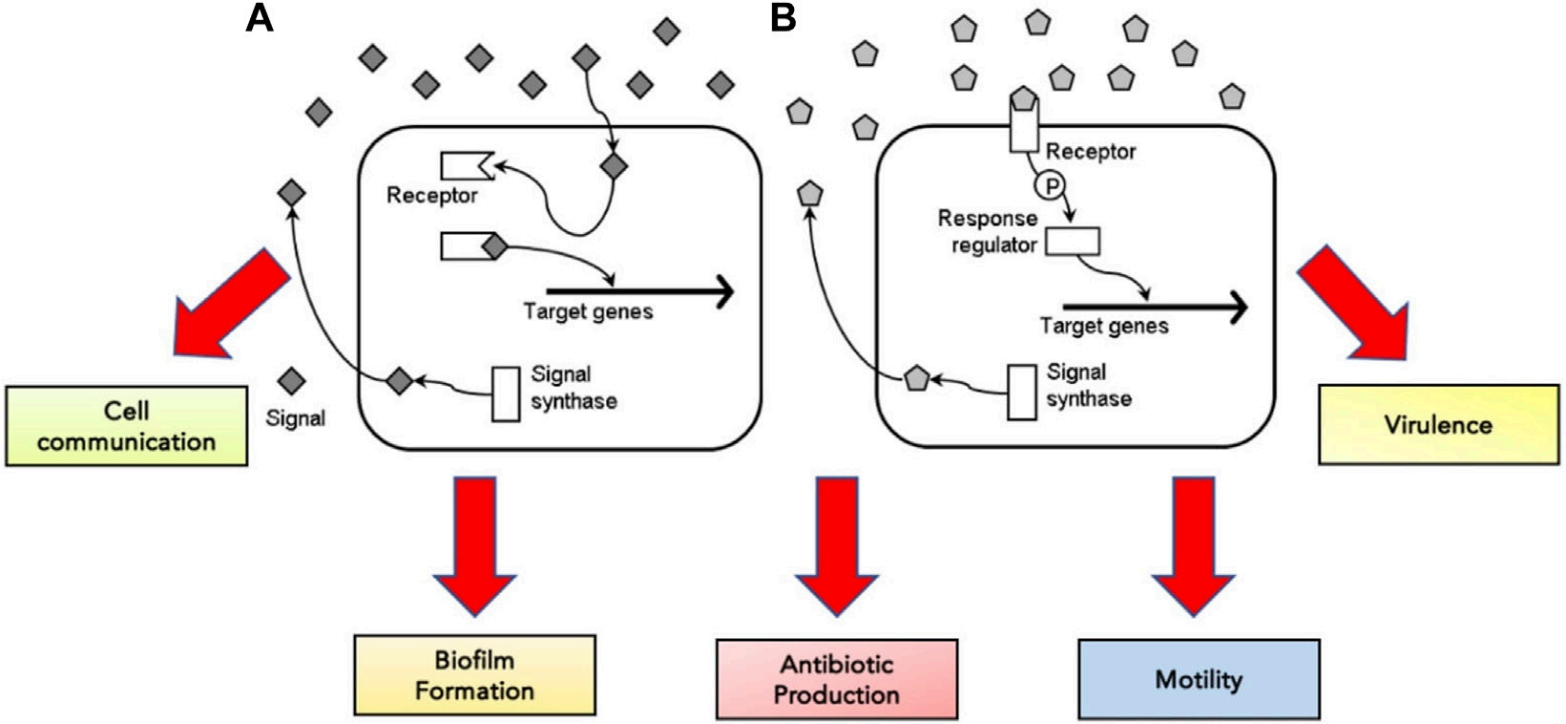
The concentration of signal molecules produced by the enzyme signal synthase is consistently monitored by bacterial cells in the surrounding environment. When signal molecules are low in concentration, the signal molecule binds to the target receptor, either in the cytoplasm **(A)** or on the cell surface **(B)**—adapted from Defoirdt et al. (2010).

Bacterial cells rely predominantly on quorum sensing for cell communication and regulating various physiological functions, including biofilm formation, antibiotic production, motility and virulence (Miller & Bassler, 2001; Defoirdt et al., 2010) (Figure 3). They release extracellular signal molecules termed autoinducers through membrane transporters upon reacting to changes in bacterial cell density. Adhesion to specific transcriptional regulators alters gene expression (Miller & Bassler, 2001; Rutherford & Bassler, 2012; Jiang et al., 2019). The target receptor through which autoinducers achieve their functions can be located in the cytoplasm or on the cell surface. If autoinducers bind to the receptors in the cytoplasm, they can control the activation and inactivation of gene transcription within the cell. In contrast, signal molecules bound to the receptors on the cell surface regulate gene transcription through phosphorylation and a response regulator (Defoirdt et al., 2010) (Figure 3).

Common quorum signals with significant roles in bacterial growth and pathogenesis include acyl-homoserine lactones (AHLs), autoinducing peptides (AIPs) and autoinducer-2 (Jiang et al., 2019). AHLs are more commonly produced by Gram-negative bacteria, while AIPs are produced predominantly by Gram-positive bacteria (Jiang et al., 2019). The involvement of quorum signaling in diverse functions suggests its significance to bacterial cells. Therefore, targeting quorum sensing with anti-quorum sensing agents can be an effective method of inhibiting biofilm production. Previous studies, for instance, have suggested that mutants of Gram-negative *P. aeruginosa* lacking a proper quorum-sensing system formed defective biofilms sensitive to detergents, paving a path for further exploration of their potential role in the development of novel antibacterial surfaces (Sakuragi & Kolter, 2007).

Recently, the application of natural and synthetic furanones as QSIs has been investigated. Furanones are naturally occurring chemicals in various marine and terrestrial plants, fungi like *Aspergillus spp*., and fermented foods (Proctor et al., 2020). Alternatively, furanones can be synthesized using chemical precursors. Natural furanones have demonstrated their ability to impair biofilm formation and reduce biofilm thickness by a great margin. For example, low concentrations of furanone have been shown to inhibit the quorum sensing mechanism without affecting cell viability in *Escherichia coli.* (Proctor et al., 2020).

Similarly, halogenated furanones have also been shown to reduce motility and biofilm production in bacterial cells (Hentzer et al., 2002; Manefield et al., 2002). Studies conducted by Chang et al. (2019) have suggested the promising nature of aromatic furanone rings, specifically in inhibiting an essential gene (*LasR*) responsible for quorum sensing in Gram-negative *P. aeruginosa*. Reportedly, aromatic furanones have been influenced by the number of rings in their structure (Park et al., 2017). Halogenated furanones have demonstrated their capacity to weaken and possibly hinder the activity of target genes responsible for biofilm production and virulence in *E*. *coli*, which are controlled explicitly by AHLs, and reduce the cytoplasmic concentration of essential transcriptional activator proteins (Manefield et al., 2002). Furanones can bind to receptors critical for cell communication *via* quorum sensing and block autoinducers’ access to these receptors (Zhou et al., 2020).

The discovery and development of antimicrobial surfaces with quorum sensing inhibitors indicate promising prospects in the application, given that the process does not necessarily require the use of antibacterial chemicals, such as antibiotics and detergents, that can cause antibacterial resistance. Since QSIs can achieve their functions below minimal inhibitory concentrations, unlike traditional antibacterial agents, they are expected to significantly lower selection pressure on bacterial cells, resulting in resistance development (Hentzer et al., 2002). Nonetheless, extensive research is needed to examine the effects of QSIs on other strains of infectious bacteria and potential risk factors, such as cytotoxicity of halogenated furanones, and establish the specific working mechanisms of QSIs in both Gram-negative and Gram-positive bacteria to further harness them for use in *in situ* environments.

### Antimicrobial peptide-based antimicrobial surfaces

Antimicrobial peptides (AMPs) have emerged as attractive candidates for the development of antimicrobial surfaces owing to their broad spectrum of activity (Alves and Olivia Pereira, 2014; Li et al., 2014), high efficacy at low concentrations and potential target specificity (Alves and Olivia Pereira, 2014). The innate immune system of most organisms consists of AMPs that offer protection against the threats of invading microorganisms, often serving as the first line of defense (Holaskova et al., 2015). A wide array of AMPs has been isolated from various animals, vertebrates and invertebrates, plants, bacteria, viruses, and fungi (Alves and Olivia Pereira, 2014). AMPs are categorized based on their secondary structure, which is constituted by β-sheet peptides stabilized by disulfide bridges, quantities of which range from two to four α-helical peptides, loop peptides formed from a single disulfide bridge and extended structures rich in amino acids like glycine, proline, tryptophan, arginine and histidine (Alves and Olivia Pereira, 2014; Mahlapuu et al., 2020).

**FIGURE 4:**
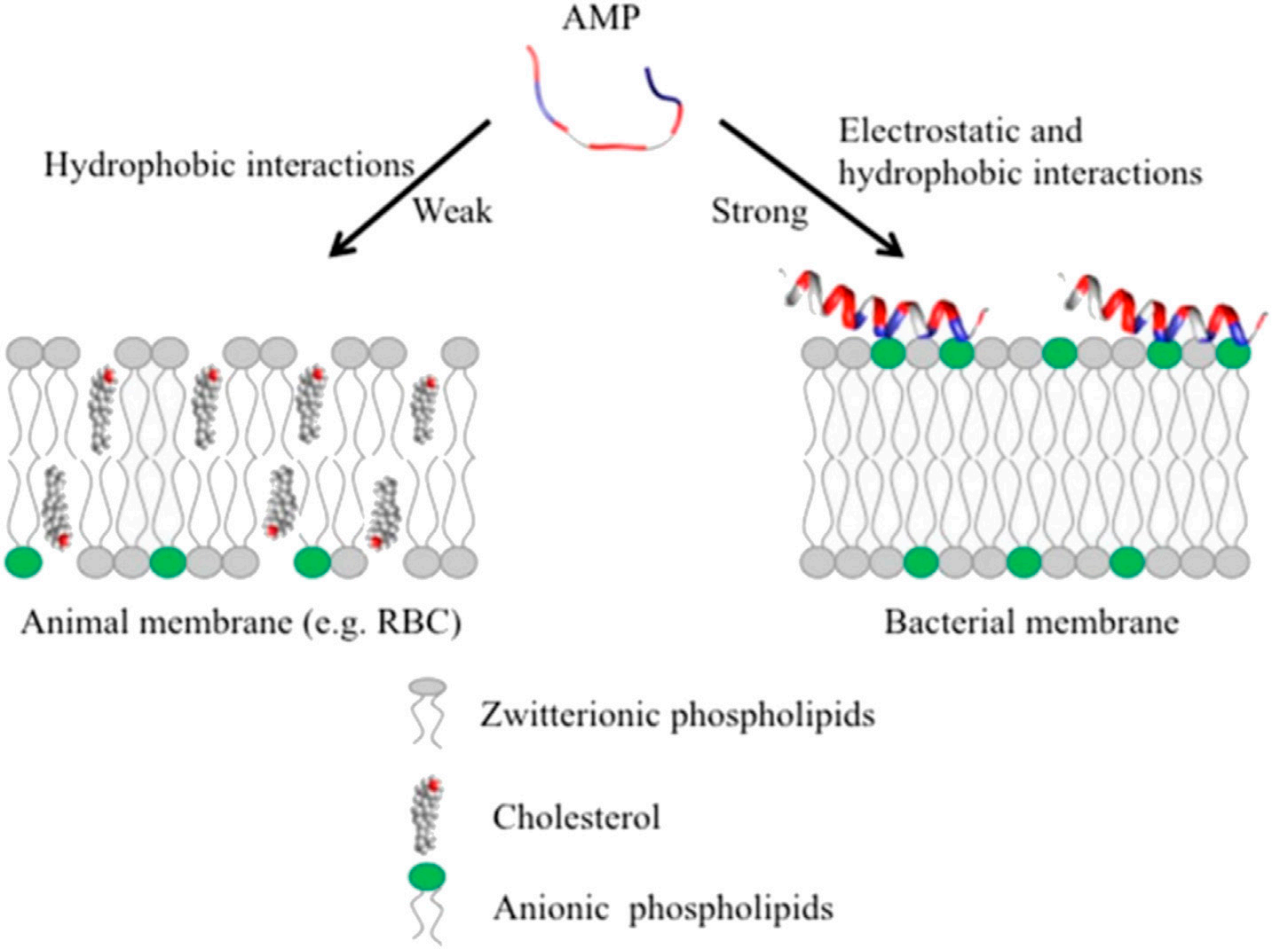
Unlike animal membranes, bacterial membranes are composed of negatively charged phospholipid head groups which attract the positive charge of AMPs, adapted from Kumar et al., 2018.

**FIGURE 5:**
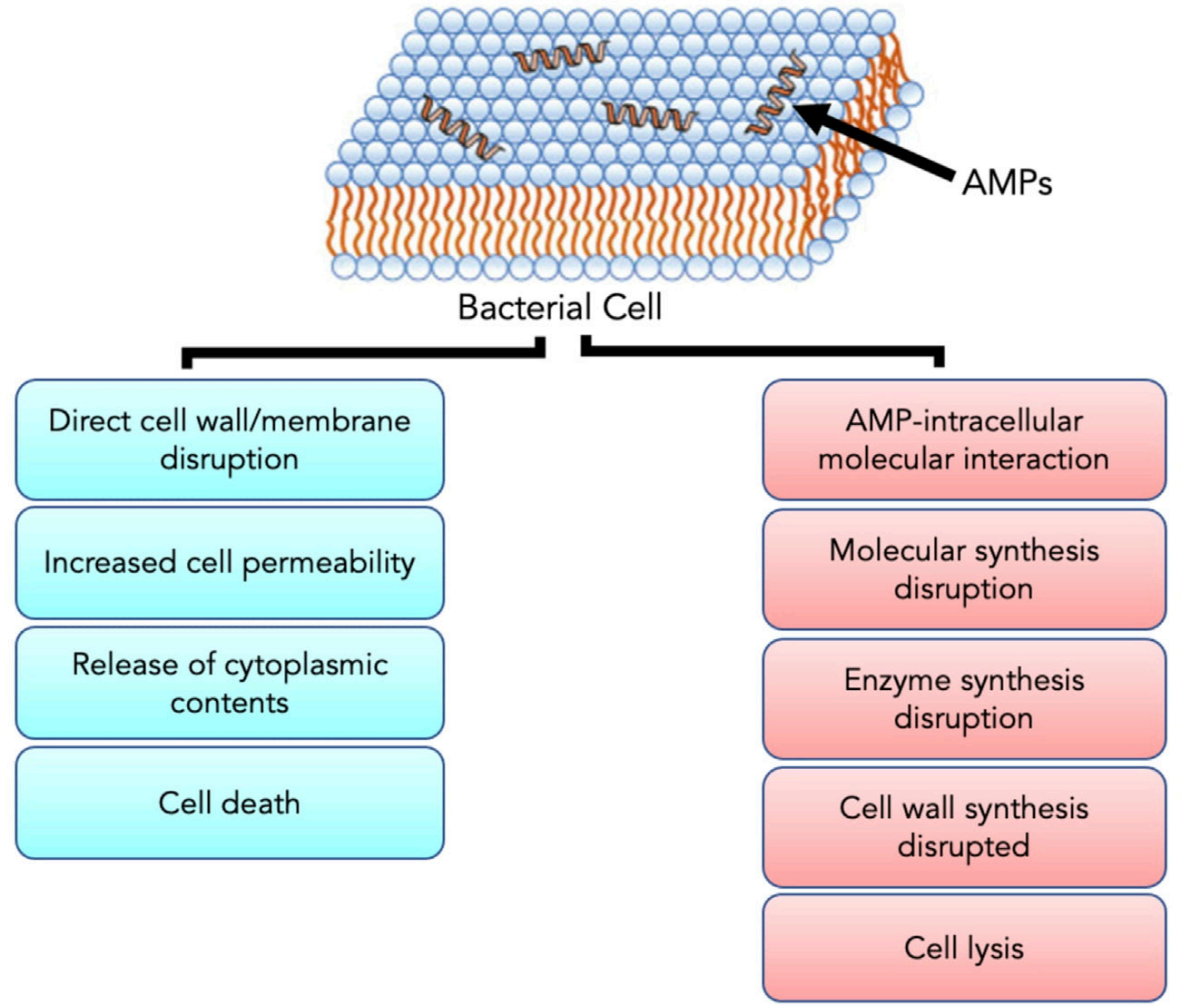
The positive charge of AMPs facilitates interaction with the negatively charged bacterial cell membrane. Upon interaction and subsequent penetration into the cell, AMPs either result in cell death through direct cell wall/membrane disruption or cell lysis by disrupting the synthesis of proteins and cell walls. Adapted from Zhang et al. (2021).

AMPs have exhibited their ability to inhibit biofilm formation, detach established biofilms and increase the vulnerability of biofilms to other antimicrobials (Alves and Olivia Pereira, 2014; Yasir et al., 2018). Most AMPs work by destabilizing bacterial membranes by either altering the thickness of the membrane or causing leakage of cell contents through membrane permeabilization (Mahlapuu et al., 2020). Although AMPs are diverse in terms of structure and function, these peptides share specific common properties that favour their application as antimicrobials; for instance, a highly cationic character, their ability to adopt amphipathic structures owing to the large proportion of hydrophobic structures in their overall architecture and their tendency to be directed to the cell membrane (Epand & Vogel, 1999; Alves and Olivia Pereira, 2014). Given that AMPs play a significant role as the first line of defense against invading pathogens, they possess intrinsic features that make AMPs promising alternatives to conventional antibiotics. AMPs exhibit cell selectivity due to their ability to differentiate host and bacterial cells, rapid mechanism of action and low propensity for developing bacterial resistance (Alves and Olivia Pereira, 2014). It is believed that their cationic nature is critical in conferring selectivity towards bacterial cells compared to mammalian cells (Epand & Vogel, 1999). Electrically neutral phospholipids can be found in the outer leaflet of the cytoplasmic membrane of eukaryotic organisms, whereas lipids with negatively charged heads lie in the inner leaflet (Shen et al., 2019). Contrarily, bacterial membranes are composed of negatively charged phospholipid head groups (Figure 4). The positive charge of AMPs facilitates them to associate with bacteria’s negatively charged cell membrane (Figure 4). The hydrophobicity of AMPs promotes their penetration into the hydrophobic interior of the cell membrane, resulting in cracks in the cell membrane and subsequent cell lysis (Wang & Vermerris, 2016) (Figure 5). Alternatively, AMPs can penetrate the cytoplasm and inhibit protein synthesis (Zhang et al., 2021) (Figure 5). Additionally, since AMPs act on the bacterial cell membrane, the propensity for developing bacterial resistance decreases substantially and the speed of action increases. Developing resistance to adapt to their environment requires the bacteria to re-structure or re-design their cytoplasmic membrane by changing the composition and organization of its constituent lipids, which can be energetically costly and, therefore, thermodynamically unfavourable (Alves and Olivia Pereira, 2014).

Antibacterial coatings have previously been created using protein immobilization techniques. Nature has drawn much inspiration to develop strategies to prevent bacterial colonization by applying AMPs. For instance, amphibians and fish secrete a dermal chemical slime containing AMPs to avoid the colonization of their skin by invading bacteria (Simmaco et al., 1998). Taking these species as a source of inspiration, physical immobilization methods, like layer-by-layer (LbL), have been developed and explored to immobilize AMPs on surfaces to create antibacterial coatings. The fundamental basis of the technique lies in the adsorption of polycations and polyanions on a solid substratum (Alves and Olivia Pereira, 2014). The approach enables AMPs to be embedded in the multilayer architecture of a rich substrate network to construct functional films. The thickness of LbL coatings, which is governed by the number of layers deposited, determines the quantity of AMPs bound to the surface, which controls the antimicrobial activity of the overall coating (Alves and Olivia Pereira, 2014). However, the layer-by-layer approach is limited to using water-soluble and highly charged AMPs. The resultant electrostatic interactions between the peptides and the poly-electrolyte matrix can potentially denature the proteins and affect the durability of the coating, compromising the antimicrobial activity of the structure (Alves and Olivia Pereira, 2014). Therefore, variations of AMPs have been created to produce functional films by incorporating insoluble AMPs with an amphiphilic polysaccharide to construct a negatively charged complex assembled using a layer-by-layer approach with positively charged homopolymers (Guyomard et al., 2008).

An alternative method of fixing AMPs on a surface identified is to covalently immobilize them through their grafting on a self-assembly monolayer (SAM) (Alves and Olivia Pereira, 2014; Acosta et al., 2019). Molecular assemblies are formed on the surface spontaneously upon adsorption onto a substrate. Humblot et al. used SAM to immobilize the peptide magainin-1 on a gold surface (Humblot et al., 2009). When tested against three Gram-positive bacteria, grafted magainin-1 could reduce bacterial adhesion by more than 50% and kill adhered cells.

Recently, nanotechnology-based techniques have also emerged to allow for the construction of nanofibers as carriers of AMPs. Recent studies have demonstrated that AMP can be electrospun into biodegradable and biocompatible polymer nanofibers. Heunis et al. (2013) incorporated AMP nisin into polylactide nanofibers in one study. The application of AMP nisin decreased the cell number of *S. aureus* by more than 99.9%. Nanofiber-based antimicrobial surfaces containing AMP have typically been created using polyvinyl alcohol (PVA) and polyethylene oxide (PEO) for loading AMP (Wang & Vermerris, 2016). In addition, the presence of hydrophobic moieties in AMPs has been revealed to facilitate AMP-based nanofiber self-assembly (Li et al., 2017).

Although the development of AMP-based antimicrobial surfaces has created many opportunities for their use in therapy, some potential challenges may need to be overcome. Firstly, microbes may resist AMPs, particularly after immobilization on surfaces, rendering them ineffective (Yasir et al., 2018). It is believed that the interactions of AMPs with biofilm and extracellular polymeric substances can induce antibacterial resistance (Otto, 2006; Nuri et al., 2015). Hence, extensive investigations on long-term interactions between AMPs and microbes are required. Secondly, some past studies have suggested the need to further enhance the selectivity of AMPs to target only bacterial cells, leaving mammalian cells unharmed (Chen et al., 2019). Electrostatic interactions are essential for AMPs to bind to bacterial cell surfaces. These interactions can neutralize negatively charged bacterial surfaces (Nuri et al., 2015). It can, thus, reduce peptide binding to the bacterial surface, reducing the efficacy of AMPs (Nuri et al., 2015). Thirdly, the effectiveness of AMPs against Gram-positive bacteria, like *S. aureus*, may not be considered due to the presence of an additional peptidoglycan cell wall in these microorganisms, which can undergo structural changes to build AMP resistance (Nizet, 2006). These bacteria are also reported to have higher minimum inhibitory concentrations than AMPs (Nizet, 2006). Recent studies have suggested that recombinant SAMs may have a more substantial anti-biofilm effect on these strains (Acosta et al., 2019). Fourthly, incorporating antimicrobial agents in nanofibers may alter their biocidal efficacy upon immobilization on a given surface. Thus, the biocidal effects of antimicrobial agents may differ notably by themselves instead of when immobilized (Wang & Vermerris, 2016). Yasir et al. have suggested through a study that the rate of action of surface-bound AMPs is lower than their free counterparts at minimum inhibitory concentrations, particularly against *P. aeruginosa* (Yasir et al., 2018). Lastly, AMPs can also be prone to degradation by enzymes (Escobar et al., 2020).

### Antimicrobial enzyme multilayer coatings

Antimicrobial enzymes have attracted much interest for their potential role in creating antimicrobial surfaces. Applying layer-by-layer enzyme immobilization techniques to create biologically stable multilayer coatings of antibacterial enzymes makes them appealing on target surfaces (Ivanova et al., 2015). Enzymes like acylase have previously been reported to exhibit an inhibitory effect on the quorum sensing capacity of bacteria. In one such study, acylase demonstrated its ability to degrade N-acyl-homoserine lactones (AHLs), critical quorum-sensing regulators, in Gram-negative bacteria (Xu et al., 2003). Without the functional autoinducers, quorum sensing in bacteria was effectively inhibited. Similarly, multilayer coatings of amylase have been effective against Gram-negative bacteria, like *P. aeruginosa*, and Gram-positive bacteria, like *S. aureus* (Ivanova et al., 2015). Ivanova et al. have reported the ability of amylase to degrade polysaccharides, which play an essential role in bacterial adhesion, to suppress biofilm formation.

The antibacterial effects of hydrogen peroxide (H_2_O_2_)-producing enzymes like glucose oxidase and cellobiose dehydrogenase (CDH) in multilayers through their immobilization between positively charged synthetic antifouling copolymers have also been studied lately (Escobar et al., 2020). Vaterrodt et al. (2016) have reported the effectiveness of CDH-based multilayers in biofilm reduction. The location of CDH in the multilayer influenced its performance. H_2_O_2_ produced by CDH diffuses and attacks the surrounding bacteria (Thallinger et al., 2016) and disrupts membrane layers, energy production and protein synthesis in bacterial cells (Nyanhongo et al., 2017). CDH combined with cellobiose is reliable for use against bacteria because cellobiose serves little to no function in microorganisms’ growth, leaving most of it converted to H_2_O_2_ (Nyanhongo et al., 2017).

Multilayer coatings of antimicrobial enzymes have great potential for use as antimicrobial surfaces. Typically, these surfaces are stable and can reduce the risk of developing antimicrobial resistance and toxicity in mammalian cells (Francolini et al., 2017). However, similar to AMP-based antimicrobial surfaces, immobilization of enzymes on surfaces may reduce their capacity to perform efficiently. Furthermore, the positioning of these enzymes on substrates can affect the system’s efficacy.

### Quaternary ammonium compounds

Quaternary ammonium compounds (QACs) are desirable due to their environmental stability, impressive cell membrane penetration properties, low toxicity and low corrosivity (Roy et al., 2008). Some of the most commonly used QACs are benzalkonium chloride (BAC), dodecyl didecyl dimethyl ammonium chloride (DDAC), and alkyl dimethyl benzyl ammonium chloride (ADBAC), which have been used in different applications and purposes ranging from surfactants to antistatic agents and as active ingredients for disinfectants and hand sanitizers. The cationic nature of the N-alkyl chain of QACs is believed to contribute to biocidal activity and lipophilicity (Beyth et al., 2015). Surfaces immobilized with QAC tend to have a highly positive surface charge, facilitating their adhesion to bacterial cells (Gottenbos et al., 2002). The association of QACs with cationic polymers offers multiple benefits; for instance, the effects of antimicrobial activity are enhanced and prolonged, residual toxicity is significantly reduced, propensity for tissue irritation in mammals is minimized, and selectivity is increased (Roy et al., 2008).

The mechanism of action of QACs is mainly based on the adsorption of positively charged QACs on the negatively charged cell surface of microorganisms by electrostatic interactions followed by their diffusion through the cell wall, which is promoted by long lipophilic alkyl chains of QACs, cell membrane binding and its subsequent disruption and loss of cytoplasmic contents (Roy et al., 2008). They can also inactivate enzymes responsible for producing energy in the bacterial cell (McKeen, 2012). QACs are known to have a better inhibitory effect on Gram-positive bacteria than Gram-negative bacteria and can perform better in the absence of mineral salts (Hegstad et al., 2010). The decrease in activity against Gram-negative bacteria results from the presence of two cellular membranes, unlike Gram-positive bacteria with one phospholipid cell membrane and one peptidoglycan cell wall (Jennings et al., 2015). Previously, studies have demonstrated bactericidal effects of QA-silica coatings on silicone rubber on *Staphylococci* and some reduction in viability of Gram-negative bacteria, like *E. coli* and *P. aeruginosa*, *in vivo* and *in vitro* (Gottenbos et al., 2002). On glass, these compounds inhibited the adhesion and growth of Gram-positive bacteria and Gram-negative *E. coli* (Song et al., 2011).

More recently, QACs have also been used to create surfaces that exhibit self-cleaning and antibacterial properties. Mussel-inspired coating technology was used to form a hydrogel attached to a membrane surface through surface crosslinking polymerization of a thermal-responsive polymer and a quaternary ammonium compound (Wang et al., 2018). As the surface superhydrophilicity could change in response to the surrounding temperature, the initial hydrophobicity of the surface first attracts bacterial cells. Once attached, the adhered bacterial cells collapse the hydrogel, revealing the QACs that kill the adhered bacterial cells while also attaining self-cleaning properties. After resuming its hydrophilicity and swelled thickness, the hydrogel resumed its function by reducing the adhesion between bacteria and the membrane surface (Wang et al., 2018). When tested against *S. aureus* and *E. coli*, the ratio of dead bacteria increased as the proportion of QAC in the hydrogel layers increased (Wang et al., 2018).

As QACs are widely used as domestic and industrial cleaning products and disinfectants in healthcare settings to disinfect hands and decontaminate surfaces (McBain et al., 2004; Smith et al., 2007; Jones & Joshi, 2021), the widespread use of QACs in clinal, household and industrial settings have created a few concerns. Though their type determines the biodegradability of QACs, these compounds generally have poor biodegradability and can remain in the environment for an extended period (Hegstad et al., 2010). The biodegradability of QACs is thought to decrease further with an increasing number of non-methyl alkyl groups (Garcia et al., 2001; Ying, 2006). Bacteria in the environment can be exposed to diluted doses of QACs, and it can cause the development of antimicrobial resistance (Jennings et al., 2015). Prolonged exposure of bacterial cells to QACs can induce selection pressure on these microorganisms. The survival of bacterial clones with higher minimum inhibitory concentration may not be affected, despite QACs (Buffet-Bataillon et al., 2012). Current literature suggests that acquired QAC resistance in Gram-positive bacteria is mediated by the negative transcriptional regulator (*QacR*), which facilitates the overexpression of the associated gene and results in the production of QAC-specific efflux pumps on the cell surface to pump out QACs (Mahoney et al., 2021). Even though QAC resistance in Gram-negative bacteria is less common, their tolerance toward QACs is believed to be higher (Mahoney et al., 2021). Nevertheless, promising novel technologies involving the combination of QACs with other materials have recently emerged as having superior bactericidal efficiency. In one such study the impregnation of QACs in anodized aluminum with nanoholes was shown to destroy a range of Gram-positive and Gram-negative bacterial cells within seconds of contact (Valiei et al., 2018). Hence, further development of QAC-based antimicrobial technologies against a wider range Gram-negative and Gram-positive bacteria could resolve the issue with QAC resistance and could potentially contribute in the ongoing efforts towards the development of efficient and reliable QAC-based antimicrobial surfaces.

## Concerns

As discussed, developing antimicrobial/antifouling surfaces has created opportunities for tackling the threat of bacterial colonization of surfaces and biofilm formation. Nonetheless, certain hurdles may need to be overcome.

Firstly, antimicrobial surfaces capable of killing bacteria may not necessarily be able to clean the debris. Thus, killing may not always entail cleaning. The accumulation of dead bacteria on antimicrobial surfaces may promote the further proliferation of bacterial cells, thereby reducing their antimicrobial effects (Wang & Vermerris, 2016). The electrostatic interactions between cationic antimicrobial agents and anionic bacterial cells that facilitate bacterial contact killing may also unfavourably contribute to biofouling once the threshold is reached (Riga et al., 2019). Therefore, there may be a need to effectively incorporate methods to clean the debris upon killing the bacteria. It can raise questions regarding the practicality or functionality of these antimicrobial or antifouling surfaces and causes the need to investigate if combining multiple systems, i.e., physically and chemically modified surfaces, may produce more practical or desirable results due to their synergistic effect. It demands a biochemical investigation of natural antimicrobial surfaces to deduce if the antimicrobial effect is merely a result of their topological or mechanical properties or combined chemical/biochemical reactions. Surface regeneration, polishing, and surface layer shedding methods are being explored to address these issues (Riga et al., 2019). A study has previously reported that surface regeneration can be achieved through hydrolysis of polycationic polymer brushes, converting them from their antimicrobial state to a non-fouling bacteria-resistant state (Cheng et al., 2008; Riga et al., 2019). The technique has exhibited a bactericidal effect on *E. coli* and released almost all dead bacteria upon hydrolysis of polymer brushes (Cheng et al., 2008). The surface polishing technique involves the degradation of polymer bases by either hydrolysis or bacterial enzymes (Riga et al., 2019).

**FIGURE 6:**
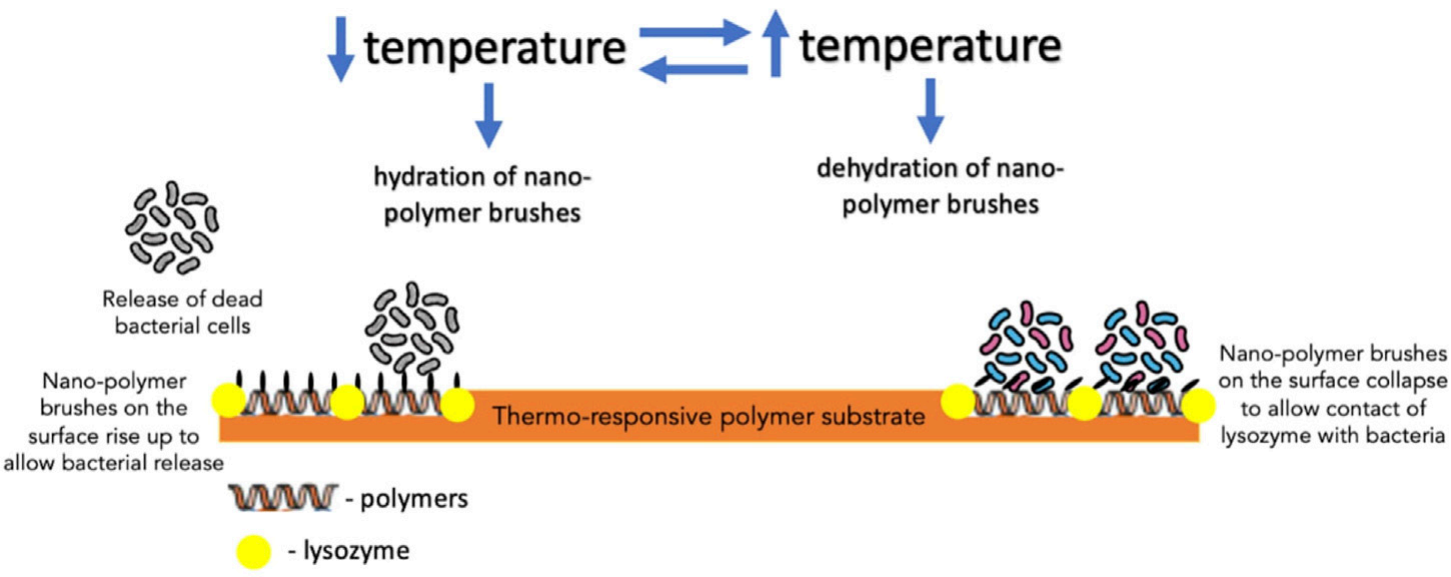
The mechanism of action of thermo-responsive polymer substrate with lysozyme embedded in the polymer-free regions of the substrate against bacteria.

**FIGURE 7:**
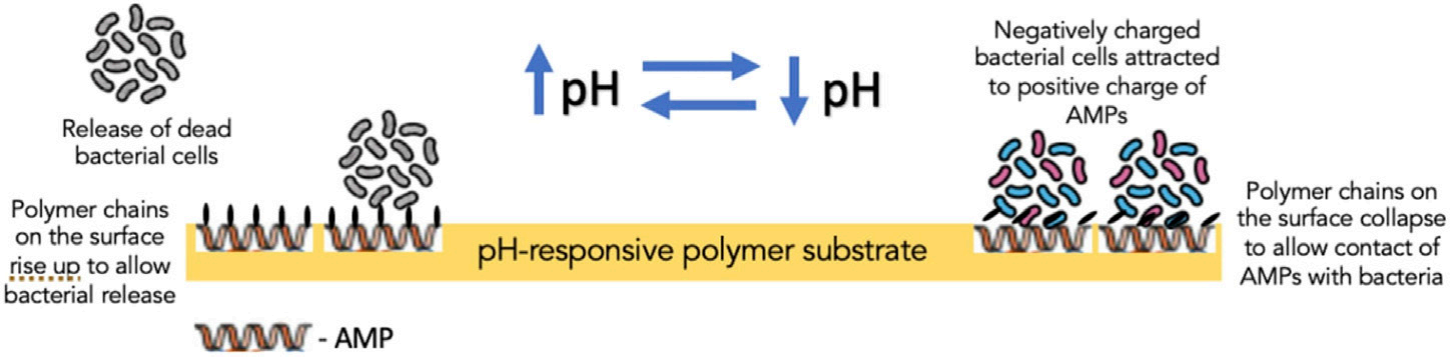
The mechanism of action of pH-responsive polymer substrate containing antimicrobial peptides in the inner layer of the substrate.

Furthermore, biocidal components, such as antimicrobial enzyme lysozyme, QACs and AMPs, in creating switchable surfaces with antifouling and bactericidal properties, have also been investigated. In one such study, lysozyme was adsorbed into the polymer-free regions of a thermally responsive polymer substrate to create a surface which, in response to the temperature of the aqueous solution, could induce changes to the conformation of the polymer brushes dispersed on the surface (Yu et al., 2014a). When dehydrated, polymer brushes could bend down to expose adsorbed lysozyme, which came into contact with bacterial cells; on the other hand, hydrated polymer brushes covered the lysozyme to block its direct contact with bacteria (Yu et al., 2014a) (Figure 6). Its effect was more profound on Gram-positive bacteria (*S. epidermidis*) than Gram-negative bacteria (*E. coli K 12*). Yan et al. reported the effect of AMPs immobilized on a polymer substrate on Gram-positive *S. aureus*. The overall structure was believed to be biocompatible as the cationic and hydrophobic nature of AMPs is protected by the hydrophilic and anionic outer layer of the pH-responsive polymer. Bacterial colonization acidified the surface, triggering polymer chains to collapse and exposing AMPs embedded in the inner layer to facilitate contact with bacterial cells and, thereby, bacterial cell disruption (Yan et al., 2016). Subsequently, as the pH increased gradually, the polymer chains regained their hydrophilicity to rise and allow bacterial release (Yan et al., 2016) (Figure 7).

The new generation of superwettable surfaces with antifouling/antimicrobial characteristics shows promising results. However, their long-term effectiveness and potential activity against other bacteria remain uncertain. Further investigation is required to better realize the effectiveness and durability of these techniques *in situ* conditions. It is also critical to examine and understand how these surfaces can trigger regulatory cascades in bacterial systems to boost further development and discern the potential effects of these methods on bacterial gene regulation and expression.

## Future applications and implications

Recent advances in techniques that allow for the fabrication of antimicrobial surfaces have created possibilities for their application in real life. Among their many potential applications, nanostructured biomaterials can be of greater significance in healthcare, the food industry and public spaces. Nanostructured surfaces can also be applied to sanitary equipment or tools.

Modern healthcare has become relatively more reliant on medical devices, such as heart valves, stents, pacemakers, contact lenses, and other prosthetic devices. Furthermore, advancements in healthcare technology have made medical implantations possible and widely accessible. The extensive use of medical implants and devices has pushed the need to exercise safer medical practices to avoid contamination of implants and devices by microorganisms to prevent internal infections. Despite numerous antimicrobial agents, healthcare-associated infections, of which more than half are associated with medical implants and devices, have been common for years (VanEpps & Younger, 2016). Environmental bacteria in the operating room and the patient’s skin can reach biomaterial implants directly during their insertion into the human body. Such a trend indicates the need to either further develop currently used methods or use new approaches to fight against pathogens more effectively, such that traditional methods can be used in combination with new approaches. If tissue integration, which is key to successful implantation, is preceded by bacterial adhesion and colonization, it can pave the way for biofilm development. The growing emergence of antimicrobial-resistant microorganisms has challenged traditional ways of overcoming infections. Moreover, biofilm-associated bacteria are more resistant to antimicrobial stressors and antibiotics. Bacteria bound to biofilms are protected from natural host defenses. Past a certain stage, medical devices often become susceptible to biofilm formation. The occupation of bioimplants and neighbouring tissues by bacteria can have life-threatening consequences. In some cases, biofilm formation can result in metastatic infection once a fragment of the biofilm detaches and the bacterial cells are carried downstream (Zhang & Wagner, 2017), leading to the development of a new biofilm.

Integrating antimicrobial surfaces/coatings with medical devices and implants can be a promising way of preventing bacterial colonization and subsequent infection. The antifouling properties of lotus leaves, shark skin and wings of cicada/dragonfly/butterfly can be mimicked by constructing micro-structures and nanostructures with similar or identical topographies and other relevant features. Antifouling surfaces repel and prevent cell surface attachment. The significant reduction in adhesion between bacteria and the nanostructured surface imposes substantial stress on bacterial cells. Studies have suggested that bacterial cell walls stretch and deform as they interact with textured surfaces, leading to cell death. Furthermore, antimicrobial coatings can also exert bactericidal effects on settling bacterial cells. The combined effects of antifouling surfaces and biocidal agents to form superwettable surfaces can ensure that surfaces can kill and self-cleanse to eliminate bacteria from the surface. Despite the promising future of nano-biomaterials as antimicrobial surfaces, the impact of micro-structured and nanostructured biomaterials in the body, in the long run, needs to be explored before their use can be promoted. Therefore, an extensive *in vivo* evaluation of the durability and stability of suggested nanostructures is required. Furthermore, the implants and devices need to be biologically compatible and non-toxic. They should also exhibit appropriate mechanical and wear-resistant properties.

The use of nanostructured surfaces with antimicrobial properties can also be extended to the food industry. The maintenance of a microbe-free environment is critical in food preparation and packaging. A wide variety of equipment and bacteria present in the atmosphere can potentially be sources of contamination. Developing equipment using antimicrobial/antifouling micro-structured and nanostructured surfaces/coatings can effectively prevent adhesion to a considerable extent and even kill the settling bacteria.
